# Disaggregated data on age and sex for the first 250 days of the COVID-19 pandemic in Bucharest, Romania

**DOI:** 10.1038/s41597-022-01374-7

**Published:** 2022-05-31

**Authors:** Marian-Gabriel Hâncean, Maria Cristina Ghiță, Matjaž Perc, Jürgen Lerner, Iulian Oană, Bianca-Elena Mihăilă, Adelina Alexandra Stoica, David-Andrei Bunaciu

**Affiliations:** 1 Department of Sociology, University of Bucharest, Panduri 90–92, Bucharest, 050663 Romania; 2 Faculty of Natural Sciences and Mathematics, University of Maribor, Koroška cesta 160, 2000 Maribor, Slovenia; 3 Department of Medical Research, China Medical University Hospital, China Medical University, Taichung 404332 Taiwan; 4 Alma Mater Europaea, Slovenska ulica 17, 2000 Maribor, Slovenia; 5 Complexity Science Hub Vienna, Josefstädterstraße 39, 1080 Vienna, Austria; 6 Department of Computer and Information Science, University of Konstanz, 78457 Konstanz, Germany; 7 HumTec Institute, RWTH Aachen University, 52062 Aachen, Germany

**Keywords:** Society, Infectious diseases, Complex networks

## Abstract

Experts worldwide have constantly been calling for high-quality open-access epidemiological data, given the fast-evolving nature of the COVID-19 pandemic. Disaggregated high-level granularity records are still scant despite being essential to corroborate the effectiveness of virus containment measures and even vaccination strategies. We provide a complete dataset containing disaggregated epidemiological information about all the COVID-19 patients officially reported during the first 250 days of the COVID-19 pandemic in Bucharest (Romania). We give the sex, age, and the COVID-19 infection confirmation date for 46,440 individual cases, between March 7^th^ and November 11^th^, 2020. Additionally, we provide context-wise information such as the stringency levels of the measures taken by the Romanian authorities. We procured the data from the local public health authorities and systemized it to respond to the urgent international need of comparing observational data collected from various populations. Our dataset may help understand COVID-19 transmission in highly dense urban communities, perform virus spreading simulations, ascertain the effects of non-pharmaceutical interventions, and craft better vaccination strategies.

## Abstract (summary)

**Table:** 

Measurement(s)	COVID-19 cases • Demographic individual-level data • Nonpharmaceutical interventions
Technology Type(s)	manual curation, checks for plausibility and completeness • manual curation, checks for plausibility, completeness, conformance • manual curation, data collection, checks for plausibility, completeness, conformance
Sample Characteristic - Organism	Homo sapiens
Sample Characteristic - Environment	Urban community
Sample Characteristic - Location	Bucharest, Romania

## Background & Summary

Since the onset of the pandemic, the volume of COVID-19 data made available to the public has been unprecedented^1^. Yet, disaggregated data are still scarce, incomplete, sometimes contradictory, and with little cross-country comparability. Various reports have substantiated the need for better data. It has already been pinpointed the sheer importance of disaggregated information by gender, age, geographic location, ethnicity, and other variables relevant in a national context, especially for the developing counties^2^. Some scholars have posited that failing to acknowledge the importance of disaggregated data on gender and sex may result in significant inequalities in access to health services^3^. Others have propounded that the current lack of COVID-19 disentangled data will increase the existing sex and gender data gaps, which in turn will increase gender disparities in the health and socioeconomic effects of the pandemic, with a negative impact on females^4^. Various experts worldwide have advocated the need to produce and standardise age-disaggregated health data to improve usability and cross-country comparison^5^. They have showcased that failing to do so results in misinterpreting the patterns of SARS-CoV-2 transmission among and beyond the cohort of children, the process of prioritizing vaccination, and the inspection of the secondary effects.

As of January 21^st^, 2022, Romania has had the second-lowest vaccination rate in Europe (41% cumulative uptake of full COVID-19 vaccination scheme)^6^. On top of that, Romania is among the top ten European countries with the highest death toll (3,106 per one million people)^7^. Located in the southeast of Romania, Bucharest is the capital city and the primary economic agglomeration of the country. Services - the most impacted sector during the COVID-19 pandemic-represent the leading supplier for the local economy^8^. With a total resident population of 1,827,390^9^, Bucharest ranks third, behind Paris and Athens, by population density, among the European Union country capital cities (7,917 persons per km^2^)^10^.

The first COVID-19 case in Bucharest (case number 12 in Romania) was officially confirmed on March 7^th^, 2020. The patient, a male, aged 49 years old, entered Bucharest by plane on February 27^th^, travelling from Rome, Italy^11^. Restrictive measures were imposed, at that time, only for travellers arriving in Romania from regions in northern Italy. Evidence suggests that returning travellers from Italy had a pivotal role in the early spread of COVID-19 in Romania^12^. As of January 21^st^, 2022, the number of positive cases in Bucharest has reached a tally of 335,498, accounting for 17% of all 1,983,670 cases reported in the country^13^.

We present the first 250 days of the coronavirus pandemic in Bucharest (between March 7^th^ and November 11^th^, 2020), comprising 46,440 COVID-19 individual cases. The dataset gives for each patient: disaggregated biological sex and age, the COVID-19 infection confirmation date, the place of residence or quarantine, and the phase (stage) corresponding to the Non-Pharmaceutical Interventions (NPIs) active in Bucharest. The official measures adopted during the first 250 days critically vary in severity: from imposing a nationwide state of emergency to almost no measures at all. The NPIs have been diverse: mask-wearing, social distancing, movement restrictions (including for people aged 65+), school closures, etc. The effect of the NPIs is likely to be conditioned on the structure of the target population, on the share of specific groups within the total population^14^, and on mobility and geographic factors^15^. In effect, we divide the first 250 days into five phases given the measures’ profile (the severity). All the information (excepting the NPIs phase variable) was procured, in an anonymized format, from the Bucharest Public Health Department (the Romanian Ministry of Health). Our study received ethical approval (Decision No. 1, September 1^st^, 2020) from the Ethics Committee of the Department of Sociology (University of Bucharest). We complied with all relevant ethical regulations.

The potential re-use of our dataset is multiple: epidemiological prevalence understanding, mathematical modelling or simulations, and public health policy design or assessment. Also, it allows for measuring the effectiveness of NPIs at the city (urban community) level, which may provide a fine-grained understanding of local health policy success. A sub-sample of our dataset has already been employed to estimate the role of age and sex in spreading COVID-19 in Bucharest^16,17^.

## Methods

The dataset comprises 46,440 COVID-19 patients officially confirmed and reported between March 7^th^ and November 11^th^, 2020, in Bucharest (before the start of the vaccination campaign). These real-world data were procured from the Bucharest Public Health Department (the BPHD), Romanian Ministry of Health. To receive the data, our team sent an official request address to the BPHD (i.e., Address No. 14870, August 18^th^, 2020) on behalf of the University of Bucharest. The BPHD provided the dataset based on approval No. NT3054E (August 28^th^, 2020) in response to our request. Our study received ethical approval (Decision No. 1, September 1^st^, 2020) from the Ethics Committee of the Department of Sociology (University of Bucharest). Before their being procured, the data were anonymized by the BPHD. The 46,440 observations (patients) correspond to an investigated time window of 250 days (March 7^th^ - November 11^th^, 2020). Subsequently, we qualitatively analysed the NPIs advanced by the public health authorities in Romania^18^ during the 250-day timeframe. Accounting for their level of severity, we derived five stringency phases. Each of the 250 days was nested in one of the five phases.

**Fig. 1:**
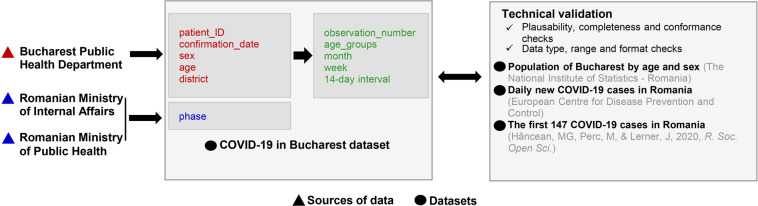
The sequence of steps taken to produce the dataset.

Figure 1 describes the steps of producing the “COVID-19 in Bucharest” dataset (or, briefly, the dataset). For each of the 46,440 COVID-19 patients, we have records of their biological sex, age, area of residence, or quarantine, as well as their official confirmation date. Each patient also has a unique code of identification (patient_ID) assigned by the BPHD. These records are marked in red in Fig. 1. Information about the NPIs corresponding to the first 250-day time interval was retrieved from official press releases uploaded on the webpage of the Romanian Ministry of Internal Affairs^18^ and from newsletters published in the “Press releases” section on the webpage of the Romanian Ministry of Public Health^19^. This information was expressed in the form of the “phase” variable (marked in blue in Fig. 1). Additionally, our team derived new variables from the original data records provided by the BPHD, i.e., observation number, age_groups, month, week, and 14-day interval. These variables are marked in green in Fig. 1. Our team performed various technical validation checks on the “COVID-19 in Bucharest” dataset (plausibility, completeness, and conformance checks). These are presented in greater detail in the “Technical validation” section. For brevity, we only mention here that during the technical validation stage, we compared the data records in our dataset to the information enclosed in other public available datasets^20–22^.

**Fig. 2:**
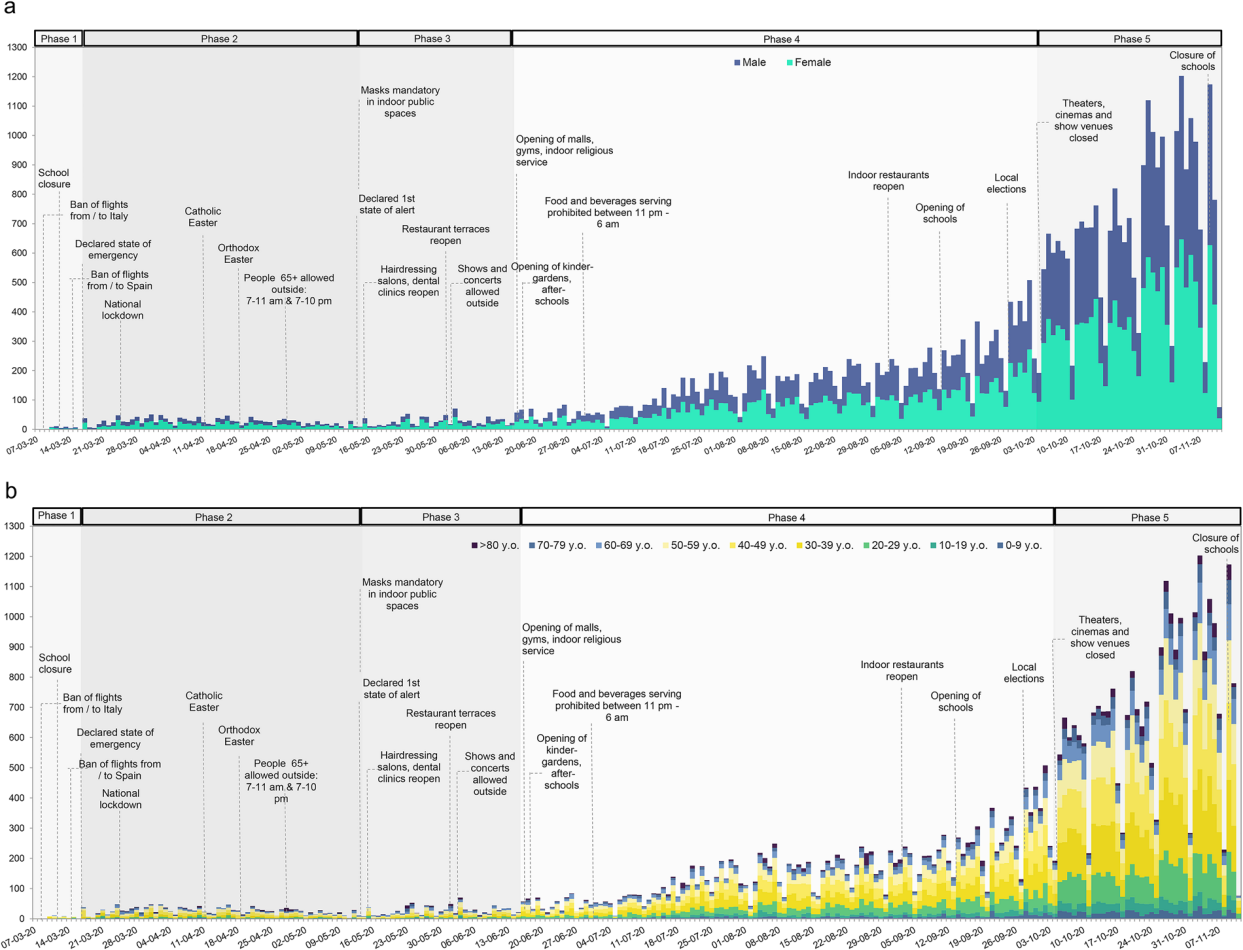
The evolution of COVID-19 confirmed cases between March 7^th^ and November 11^th^, 2020, in Bucharest, Romania. We illustrate the new daily cases by sex (**a**) and age-groups (**b**) while accounting for the stringency phases. The rendered information represents the authors’ contribution.

We used the severity degree of the NPIs to delineate the stringency phases. In Fig. 2, for informative and illustrative purposes, we represent the evolution of the COVID-19 cases in a longitudinal fashion (between March 7^th^ and November 11^th^, 2020) by major NPIs, stringency phases, sex, and age groups. The stringency phases are displayed in temporal order. The information displayed in the main content and Fig. 2 represents the authors’ contribution.

Stringency “Phase 1” ranges between March 7^th^ and March 15^th^, 2020, and corresponds to “low to moderate measures.” Forty-seven COVID-19 cases are confirmed during this phase. The major NPIs until March 15^th^ are: restriction of outdoor or indoor public and private events to 1,000 participants (March 8^th^), cessation of flights (March 9^th^), bus rides (March 10^th^), and railway travel (March 12^th^) to and from Italy, suspension of face-to-face classes (March 11^th^) in all pre-university level schools and some universities, limitation of the number of participants in indoor cultural, scientific, religious and sports activities to 50 (March 11^th^). “Phase 2”, matching the most severe measures, corresponds to “the state of emergency in Romania” and ranges between March 16^th^ and May 14^th^, 2020. One thousand five hundred fifty-one cases are confirmed amid these dates. The preliminary NPIs adopted during the state of emergency are: an extension of the suspension of in-person classes; permission of only takeaway and delivery services in restaurants and shopping malls; closure of hotels and clubs; prohibition of indoor cultural, scientific, religious, and sports events; the restriction of outdoor personal events to 100 participants; the cessation of flights to and from Spain. On March 24^th^, the military is deployed to help enforce a national lockdown. Movement outside the household is generally prohibited for non-essential purchases, with persons over 65 having their outdoor activity restricted, at first, to a two-hour interval (March 25^th^) and, then, to a three-hour interval (March 29^th^). Starting April 27^th^, non-essential movement outside the household is permitted for persons above the age of 65 both in the morning (7–11 am) and during the evening (7–10 pm).

“Phase 3”, with moderate measures, ranges between May 15^th^ and June 16^th^, 2020. Nine hundred thirty-two cases are confirmed during this interval. May 15^th^ marks the onset of the first “state of alert”, allowing hairdressing salons, barbershops, and dental clinics to reopen. Facial masks generally become mandatory in indoor public spaces, public transportation included. From June 1^st^, gradually, some of the movement restrictions are lifted, outdoor concerts and cultural events are permitted, and restaurant terraces reopen. “Phase 4”, with 18,499 confirmed cases, is a phase of the least stringent measures, ranging between June 17^th^ and October 6^th^, 2020. Since June 17^th^, shopping malls, public nurseries, and pre-schools are allowed to reopen. Pre-university level schools reopen on September 14^th^. Local elections are held on September 27^th^, with a participation rate of 37% of the 18+ Bucharest population^23^. For the entire phase, facial masks remain mandatory. “Phase 5”, with low to moderate measures, ranges between October 7^th^ and November 11^th^, 2020. Authorities confirm 25,274 COVID-19 cases during this phase. As of October 7^th^, theatres, cinemas, and show venues are closed in Bucharest, while restaurants are restricted to outdoor service only. Opening hours of stores are limited, and a night curfew is being imposed. Starting November 9^th^, all in-person classes are suspended. Masks are mandatory in both outdoor and indoor places, working spaces included. Public and private gatherings are cancelled. Table 1 displays the major NPIs by stringency phase and time interval.

**Table 1:** GANTT table displaying the major Non-Pharmaceutical Interventions during the first 250 days of the COVID-19 pandemic in Bucharest.

Categories of measures	Measures	Time interval	Corresponding stringency phases				
			1	2	3	4	5
International travel controls	Ban of flights from/to Italy	Mar. 9^th^–Jun. 23^rd^					
	Ban of bus rides from/to Italy	Mar. 10^th^–May 22^nd^					
	Ban of flights from/to Spain	Mar. 16^th^–Jul. 7^th^					
Service restrictions	Hotel closure	Mar. 16^th^–May 14^th^					
	Night club closure	Mar. 16^th^–*Nov.11*^*th* a^					
	Closure of hairdressing salons, barbershops & dental clinics	Mar. 16^th^–May 14^th^					
	Takeaway only & delivery services (in restaurants & malls)	Mar. 16^th^–Jun. 16^th^					
	Closure of restaurants & coffee shops	Mar. 17^th^–Jun. 15^th^					
	Restaurants restricted to outdoor service	Oct. 7^th^–*Nov. 11*^*th*^					
	Opening hours of stores are limited	Oct. 7^th^–*Nov. 11*^*th*^					
Stay home order	National lockdown	Mar. 24^th^–May 14^th^					
	Lockdown enforced by the army	Mar. 24^th^–May 14^th^					
	Night curfew	Oct. 7^th^–*Nov. 11*^*th*^					
Internal movement restrictions	Prohibition of movement^b^	Mar. 16^th^–May 14^th^					
	Outdoor activity restrictions for people aged 65+^c^	Mar. 25^th^–Mar. 28^th^					
	Adjusted outdoor activity restrictions for people aged 65+^d^	Mar. 29^th^–Apr. 26^th^					
	Adjusted outdoor activity restrictions for people aged 65+^e^	Apr. 27^th^–May 14^th^					
Facial coverings	Facial masks and gloves are mandatory^f^	May 14^th^–*Nov. 11*^*th*^					
Events & gatherings	Restriction of outdoor activities and events^g^	Mar. 8^th^–Mar.10^th^					
	Restriction of indoor and private events to 1,000 participants	Mar. 8^th^–Mar. 10^th^					
	Adjusted restriction of indoor events^h^	Mar. 11^th^–Mar. 16^th^					
	Restriction of outdoor activities and events to 100 participants	Mar. 11^th^–Mar. 21^st^					
	Prohibition of indoor religious events	Mar. 16^th^–Jun. 16^th^					
	Prohibition of indoor sports events	Mar. 16^th^–Jun. 1^st^					
	Restriction of gatherings of more than three persons	Mar. 22^nd^–May 31^st^					
	Prohibition of indoor cultural & scientific events	Mar. 16^th^–Aug. 31^st^					
	Re-closure of theatres, cinemas, and show venues	Oct. 7^th^–*Nov. 11*^*th*^					
School closure	Suspension of face to face university classes	Mar. 11^th^–*Nov. 11*^*th*^					
	Suspension of face to face pre-university classes	Mar. 11^th^–Sep. 14^th^					
	Suspension of face to face pre-university classes	Nov. 9^th^–*Nov. 11*^*th*^					

^a^November 11^th^ is not the date until a measure is effective, but it is the last day in our dataset. ^b^Movement outside the household is prohibited for non-essential purchases. ^c^Outdoor activity for persons over 65 is restricted to a two-hour interval. ^d^Outdoor activity for persons over 65 is limited to a three-hour interval. ^e^Non-essential movement outside the household is permitted for persons above the age of 65, both in the morning (7–11 am) and during the evening (7–10 pm). ^f^Facial masks and gloves are mandatory in public indoor spaces (public transportation included). ^g^Restriction of outdoor activities and events to 1,000 participants. ^h^Limitation of the number of participants in indoor cultural, scientific, religious, and sports activities to 50. The information illustrated in the table represents the authors’ contribution.

## Data Records

We deposited a copy of the dataset (i.e., the Bucharest COVID-19 dataset) to the generalist repository figshare^24^. Data are available in an Excel file format (.xlsx), facilitating importation into various statistical software programs such as R, Python, SPSS, Stata, SAS, or conversion to comma-separated value format (.csv). In the database, the rows designate unique individual observations (COVID-19 confirmed cases). Each observation is assigned a number from 1 to 46,440 (the total number of cases) and ascendingly ordered. Overall, the dataset renders information about all the officially COVID-19 confirmed individual cases in Bucharest, since the onset of the pandemic in the city, on March 7^th^, till November 11^th^, 2020. Specifically, we give information on the COVID-19 confirmation date and each patient’s sex, age, and geographical (administrative) location (the administrative district in Bucharest). We also give the patients’ ID codes as provided by the BPHD. These ID codes are helpful for joining (linking) this dataset to other available datasets^16,17^. Additionally, we derive four new variables from the original information. Namely, based on its COVID-19 confirmation date, each observation is nested in a (calendar) month, week, 14-day interval, and stringency phase. We make available the dataset in the English language to increase its international usage by health professionals, policy-makers, scientists, and other interested parties. We use self-explanatory and straightforward variable labels and values for users’ convenience. Also, we mark missing data by “NA” codes. We continue this section with a detailed description of all variables (data fields) available in the dataset (Table 2).

**Table 2:** The data fields (variables) included in the Bucharest COVID-19 dataset.

No	Category	Data field (variable labels)	Data format
1	Observation number	observation_number	Number (e.g., “0”, “1”, “2”, …)
2	Identification	patient_ID^a^	Number (e.g., “12”, “14”, “16”, …)
3	Epidemiological	confirmation_date^a^	Date (MM-DD-YYYY)
4		month^b^	Text (e.g., “March”, “April”, “May”…)
5		week^b^	Alpha-numerical text (e.g., “w10”, “w11”, …)
6		14_day_interval^b^	Alpha-numerical text (e.g., “w10_w11”, “w12_w13”, …)
7	Demographic information	sex^a^	Text (i.e., “male”, “female”)
8		age^a^	Number (e.g., “0”, “1”, “2”, …)
9		age_groups^b^	Alpha-numerical text (e.g., “0–4 y.o.”, “5–9 y.o.”, …)
10		district^a^	Alpha-numerical text (e.g., “Bucharest”, “District_1”, “District_2”, etc.)
11	Health policy	stringency_phase^c^	Alpha-numerical text (e.g., “phase_1”, “phase_2”, …)

Data sources: ^a^Bucharest Public Health Department, ^b^Authors, using the information retrieved from the Bucharest Public Health Department, ^c^Authors, using the information retrieved from the Romanian Ministry of Internal Affairs.

### Observation_number

There are 46,440 unique data entries (observations or patients) in the dataset. Therefore, the variable takes values from 1 to 46,440. The observation numbers are ascendingly sorted. These numbers do not reflect epidemiological progression and should not be used for that purpose. Instead, this variable uniquely identifies each observation in the dataset.

### Patient_ID

The BPHD had assigned each COVID-19 patient a numerical code due to the anonymization process. We render these patient ID codes as it is useful for joining (linking) the dataset to other available datasets^16,17^.

### Confirmation_date

For each patient, this represents the official date of test response (i.e., COVID-19 confirmation date). The dataset includes only positive tests. A number of 137 cases are reported with missing confirmation dates (marked by NA). These missing data account for 0.3% of all 46,440 patients. We record the time related to the confirmation dates in the “MM/DD/YYYY” format (“month, day, year”, in left-to-right writing direction), e.g., “03/07/2020”. The maximum number of cases confirmed in a day is 1,203 (on November 3^rd^, 2020). Between March 7^th^ and November 11^th^, 2020, there are 250 days (confirmation dates) in which the authorities report positive cases.

### Sex

This represents the biological sex of the tested individuals (patients). The dataset comprises 24,696 female patients (53%) and 21,744 male patients (47%). We report no missing cases on this variable. The variable takes two values: “male” and “female”.

### Age

This variable captures the equivalent of age in completed years, with values ranging from 0 (less than one year of age) to 101. The average value is 43.4, the median is 43.0, and the most frequent value within the dataset is 52.0. The standard deviation is 17.7. Thirty-six cases have missing data (these represent less than 0.1% of all cases). In the dataset, we mark missing values by NA.

### Age_groups

We recode the “age” variable into five-year age groups (brackets), with categories ranging from 0–4 y.o. to 85+ y.o. We derive a total of 18 groups. The category with the highest absolute frequency is the 40–44 y.o. group (namely, 5,650 positive cases correspond to this age group, accounting for roughly 12% of all data entries). Thirty-six patients have missing information (these represent less than 0.1% of all cases). In the dataset, we mark missing values by NA.

### District

**Fig. 3:**
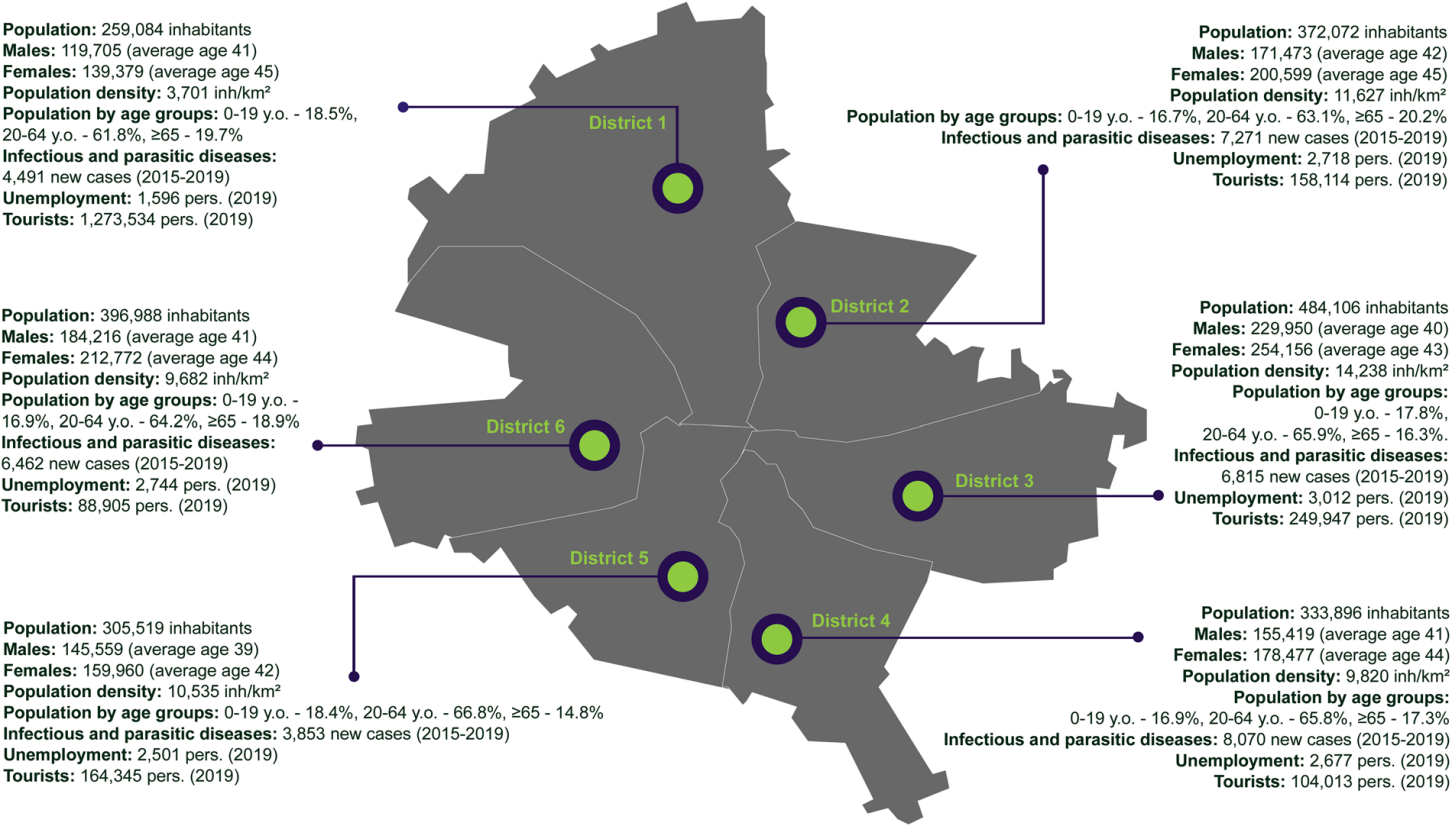
The administrative organization of the Municipality of Bucharest into six districts.

This variable refers to the six administrative units (or sectors) forming the Municipality of Bucharest, each governed by a mayor. The six districts have a clockwise geographic arrangement. For instance, District 1 is located in the north, District 4 in the south, and District 6 in the west of the city (Fig. 3). The exact district is not available for 8,043 cases (that is 17.3% of all the data entries). For these cases, we add a generic location – “Bucharest”. The “district” marks the place of residence or quarantine for a specific individual case. Due to disclosure reasons, the BPHD did not disentangle the residence from the quarantine place. The variable takes the following values: “District_1”, “District_2”, “District_3”, “District_4”, “District_5”, “District_6”, and “Bucharest”.

### Month

We derive this variable from the “confirmation_date” variable. We assign each “confirmation_date” to a “month” (each date is nested in a month). Consequently, we obtain nine months of investigation (March – November 2020). Two of these months are incomplete: March and November 2020. Further, October has the highest absolute frequency of observations (19,429 cases accounting for 41.8% of all cases). Also, in the dataset, we have 137 data entries with missing data that are marked by NA. The variable takes the following values: “March”, “April”, “May”, “June”, “July”, “August”, “September”, “October”, and “November”.

### Week

We derive this variable from the “confirmation_date” variable. We assign each “confirmation_date” to a “week” (each date is nested in a week). The variable takes as values the week number (e.g., w10, w11, w12, …, w46). Week counting starts from the beginning of the year (2020) – the first week of 2020 is January 1st – January 4th. We consider each week begins with Sunday. In our dataset, the pandemic onset in Bucharest is in week 10 (i.e., w10). The largest number of reported cases is in week 45 (6,105 observations accounting for 13% of all cases). Also, in the dataset, we have 137 data entries with missing data that are marked by NA.

### 14_day_interval

We derive this variable from the “confirmation_date” variable. We assign each “confirmation_date” to a 14-day-time-interval or two-week time window (each date is nested in a two-week time interval). We build this variable for potential analysis purposes. The variable takes as values week numbers (e.g., “w10_w11”, “w12_w13”, “w14_w15”, “w16_w17”, …, “w44_w45”, “w46_w47”). We showcase that “w10_w11” and “w46_w47” (the beginning and the end of the time window) are incomplete. We notice that the minimum number of unique COVID-19 confirmed cases (i.e., 40 representing less than 0.1% of all observations) is in weeks 10–11. Also, the maximum number of cases is in weeks 44–45 (12,045 accounting for 26% of all observations). We have 137 data entries with missing data marked by NA in the dataset.

### Stringency_phase

We derive this variable from the “confirmation_date” variable. We assign each “confirmation_date” to a “stringency phase” (each date is nested in a phase). This variable takes the following values: “phase_1”, “phase_2”, “phase_3”, “phase_4”, and “phase_5”. We define these stringency phases based on the various NPIs adopted by the authorities. These five phases are consistently different in terms of the stringency of the adopted measures. “Phase_1” covers nine days: between March 7^th^ (the first confirmed case in Bucharest) and March 15^th^ (the last day before the state of emergency). “Phase_2” corresponds to a 60-day time window between March 16^th^ and May 14^th^ (the entire period of the state of emergency). “Phase_3” covers a time frame of 33 days between May 15^th^ and June 16^th^ (the first state of alert). “Phase_4” has a 112-day duration equivalent to the period of relaxation (between June 17^th^ and October 6^th^). “Phase_5” covers 36 days and corresponds to new restrictive measures (October 7^th^ and November 11^th^). Also, in the dataset, we have 137 data entries with missing data that are marked by NA.

## Technical Validation

Before being transferred to our team, the data were collected, curated, and anonymized by the Bucharest Public Health Department (BPHD), The Romanian Ministry of Health. The BPHD performed data anonymization to solely safeguard personal data and not to infringe on the data reliability or correctness and comprehensiveness. The BPHD is a Romanian public authority mandated to develop public health policies and programs, devise preventive measures and collect public health statistics. In this regard, the BPHD is a reliable official source of data. The authors do not have details of the collection of the epidemiological data and, therefore, cannot assess the reliability of the dataset acquired from the BPHD. After receiving the dataset from the BPHD, the authors performed additional checks to ensure the technical quality. Firstly, we implemented plausibility checks, looking for duplicate cases. We identified 118 duplicate cases - with identical values on all variables. These cases were eventually removed from the dataset. Secondly, we performed completeness checks. Namely, we closely examined the variables in searching for unavailable information. We detected less than 0.3% missing values in relation to two variables, i.e., confirmation_date and age (specifically, 137 and 36 cases, respectively). We did not employ any imputation technique for these missing observations. Subsequently, we marked the missing data with “NA”.

Thirdly, we executed conformance checks and compared the information embedded in our dataset to available alternative data. To that end, we deployed three comparisons. We compared the Bucharest COVID-19 dataset against the first 147 disaggregated records officially announced by the health authorities at the onset of the pandemic in Romania^12,22^. For each of the 147 records, the authorities provided various pieces of information: the confirmation date, patients’ age, sex, residence, probable contacts, and travel history. A human-to-human network analysis of these first 147 records is available in the literature^12^. Further, we compared our dataset to the total population of COVID-19 cases officially reported in Romania for the same time window (March 7^th^ - November 11^th^, 2020). We illustrate the two-time series in Table 3. Also, we compared our dataset to the most recently updated data structure of the resident population in Bucharest (as of July 1^st^, 2020)^20^. Tables 4 and 5 illustrate the comparison between the Bucharest COVID-19 dataset and the structure of the Bucharest resident population. Eventually, we compared the stringency phase variable from our dataset to the stringency index developed by the University of Oxford^25^.

**Table 3:** The distribution of the COVID-19 confirmed cases in Romania vs the distribution of the COVID-19 confirmed cases in Bucharest, by weeks and phases, between March 7^th^ and November 11^th^, 2020.

Time interval	COVID-19 cases Romania	COVID-19 cases Romania (%)	COVID-19 cases in Bucharest	COVID-19 cases in Bucharest (%)	% differences
Weeks 10–11	83	0.0	40	0.1	0.1
*Phase 1 (07.03*–*15.03.)*	107	*0.0*	47	*0.1*	*0.1*
Weeks 12–13	1,203	0.4	321	0.7	0.3
Weeks 14–15	4,175	1.3	459	1.0	−0.3
Weeks 16–17	4,950	1.6	403	0.9	−0.7
Weeks 18–19	4,394	1.4	312	0.7	−0.7
Weeks 20–21	2,901	0.9	253	0.5	−0.4
*Phase 2 (16.03*–*14.05.)*	*15,889*	*5.1*	1,551	*3.3*	*−1.8*
Weeks 22–23	2,391	0.8	459	1.0	0.2
Weeks 24–25	3,297	1.0	485	1.0	0.0
*Phase 3 (15.05*–*16.06.)*	*6,163*	*2.0*	932	*2.0*	*0.0*
Weeks 26–27	4,766	1.5	681	1.5	0.0
Weeks 28–29	7,636	2.4	1,059	2.3	−0.1
Weeks 30–31	15,084	4.8	1,974	4.3	−0.5
Weeks 32–33	17,160	5.5	2,167	4.7	−0.8
Weeks 34–35	16,422	5.2	2,426	5.2	0.0
Weeks 36–37	16,607	5.3	2,677	5.8	0.5
Weeks 38–39	18,608	5.9	3,437	7.4	1.5
Weeks 40–41	29,203	9.3	6,307	13.6	4.3
*Phase 4 (17.06*–*06.10.)*	*115,326*	*36.7*	18,499	*40.0*	*3.3*
Weeks 42–43	52,146	16.6	8,538	18.4	1.8
Weeks 44–45	86,030	27.4	12,045	26.0	−1.4
Weeks 46–47	27,233	8.7	2,260	4.9	−3.8
*Phase 5 (07.10*–*11.11.)*	*176,804*	*56.2*	25,274	*54.6*	*−1.6*
Total cases	314,289	100.0	46,303	100.0	—

Calculations are made per time slot (weeks and phases, respectively). Percentages are calculated per column. Percentage (%) differences represent the difference between percentages in the Bucharest COVID-19 dataset and percentages in the population of COVID-19 cases in Romania. Out of the 46,440 cases, 46,303 have complete information on the infection confirmation date variable and served for recoding purposes (column “COVID-19 cases in Bucharest”).

**Table 4:** The resident population of Bucharest (as of July 1^st^, 2020) vs the COVID-19 confirmed cases in Bucharest (March 7^th^ - November 11^th^, 2020) by sex and age groups.

Variables		Population (freq.)	Population (%)	COVID-19 cases (freq.)	COVID-19 cases (%)	% differences
Sex	male	850,009	46.5	21,744	46.8	0.3
	female	977,381	53.5	24,696	53.2	−0.3
Age groups	0–4 y.o.	104,442	5.7	721	1.5	−4.2
	5–9 y.o.	90,278	4.9	685	1.5	−3.4
	10–14 y.o.	84,233	4.6	932	2.0	−2.6
	15–19 y.o.	63,034	3.4	1,134	2.4	−1.0
	20–24 y.o.	60,358	3.3	2,466	5.3	2.0
	25–29 y.o.	101,564	5.6	3,706	8.0	2.4
	30–34 y.o.	187,932	10.3	5,117	11.0	0.7
	35–39 y.o.	171,609	9.4	5,007	10.8	1.4
	40–44 y.o.	170,107	9.3	5,650	12.2	2.9
	45–49 y.o.	129,265	7.1	4,621	10.0	2.9
	50–54 y.o.	141,315	7.7	5,048	10.9	3.2
	55–59 y.o.	86,922	4.8	2,863	6.2	1.4
	60–64 y.o.	117,673	6.5	2,610	5.6	−0.9
	65–69 y.o.	114,128	6.2	2,125	4.6	−1.6
	70–74 y.o.	78,439	4.3	1,369	2.9	−1.4
	75–79 y.o.	48,021	2.6	864	1.9	−0.7
	80–84 y.o.	41,343	2.3	772	1.7	−0.6
	85+ y.o.	36,727	2.0	714	1.4	−0.6
	NA	—	—	36	0.1	0.1
	Total	1,827,390	100.0	46,440	100.0	—

Percentages (%) are calculated per column. Percentage (%) differences represent the difference between percentages in the Bucharest COVID-19 dataset and percentages in the resident population of Bucharest.

**Table 5:** The resident population of Bucharest (as of July 1^st^, 2020) vs the COVID-19 confirmed cases in Bucharest (March 7^th^ - November 11^th^, 2020) by sex and age combined.

Sex	Age groups	Population (freq.)	Population (%)	COVID-19 cases (freq.)	COVID-19 cases (%)	% differences
Male	0–4 y.o.	53,759	6.3	416	1.9	−4.4
	5–9 y.o.	46,663	5.5	326	1.5	−4.0
	10–14 y.o.	43,412	5.1	474	2.2	−2.9
	15–19 y.o.	32,799	3.9	616	2.8	−1.1
	20–24 y.o.	30,367	3.6	1,170	5.4	1.8
	25–29 y.o.	47,051	5.5	1,679	7.7	2.2
	30–34 y.o.	88,417	10.4	2,538	11.7	1.3
	35–39 y.o.	84,526	9.9	2,397	11.0	1.1
	40–44 y.o.	83,951	9.9	2,649	12.2	2.3
	45–49 y.o.	62,634	7.4	2,048	9.5	2.1
	50–54 y.o.	66,487	7.8	2,194	10.1	2.3
	55–59 y.o.	39,319	4.6	1,288	5.9	1.3
	60–64 y.o.	50,090	5.9	1,278	5.9	0.0
	65–69 y.o.	47,906	5.6	1,054	4.8	−0.8
	70–74 y.o.	31,409	3.7	663	3.0	−0.7
	75–79 y.o.	17,134	2.0	399	1.8	−0.2
	80–84 y.o.	13,226	1.6	296	1.4	−0.2
	85+ y.o.	10,859	1.3	236	1.1	−0.2
	NA	—	—	23	0.1	—
	Total	850,009	100.0	21,744	100.0	0.0
Female	0–4 y.o.	50,683	5.2	305	1.2	−4.0
	5–9 y.o.	43,615	4.5	359	1.5	−3.0
	10–14 y.o.	40,821	4.2	458	1.8	−2.4
	15–19 y.o.	30,235	3.1	518	2.1	−1.0
	20–24 y.o.	29,991	3.1	1,296	5.2	2.1
	25–29 y.o.	54,513	5.5	2,027	8.2	2.7
	30–34 y.o.	99,515	10.2	2,579	10.4	0.2
	35–39 y.o.	87,083	8.9	2,610	10.6	1.7
	40–44 y.o.	86,156	8.8	3,001	12.2	3.4
	45–49 y.o.	66,631	6.8	2,573	10.4	3.6
	50–54 y.o.	74,828	7.6	2,854	11.6	4.0
	55–59 y.o.	47,603	4.9	1,575	6.4	1.5
	60–64 y.o.	67,583	6.9	1,332	5.4	−1.5
	65–69 y.o.	66,222	6.8	1,071	4.3	−2.5
	70–74 y.o.	47,030	4.8	706	2.9	−1.9
	75–79 y.o.	30,887	3.2	465	1.9	−1.3
	80–84 y.o.	28,117	2.9	476	1.9	−1.0
	85+ y.o.	25,868	2.6	478	1.9	−0.7
	NA	—	—	13	0.1	—
	Total	977,381	100.0	24,696	100.0	0.0

Percentages (%) are calculated per column. Percentage (%) differences represent the difference between percentages in the Bucharest COVID-19 dataset and percentages in the resident population of Bucharest.

Supplementary, we deployed data type checks to ensure that the data entered had the correct data type. For instance, we examined whether the age variable contains only numerical values. Further, we ran a range check to verify whether the values taken by our variables fall within a predefined range. For example, whether the age variable has taken a reasonable range of values. Finally, we performed a format check to ensure that our variables had the predefined format. For instance, we assessed whether the values taken by the confirmation_date variable are all stored in the same fixed format, i.e., MM/DD/YYYY.

## Usage Notes

Our data records illustrate the COVID-19 prevalence in an urban community (Bucharest) for the first 250 days by providing a high-level granularity dataset. Precisely, the dataset comprises individual-level covariates, such as the age and sex of the officially confirmed patients, in a longitudinal (daily) fashion. We hope to make a contribution to the current international efforts of coalescing disaggregated empirical evidence on the spread of COVID-19. Our dataset may prove a valuable instrument for public health experts, policy-makers, scientists, and even journalists interested in assessing and better understanding COVID-19 spread in urban communities, especially before introducing the vaccines. For example, our data may demonstrate its utility in informing the efforts of scientists to statistical model or simulate the spread of diseases, in general, and of respiratory viruses, in particular. Our disaggregated observations may assist public health experts in building a comprehensive picture of the epidemiological situation. Moreover, it may help scientists establish (confirm) causal inferences in virus circulation patterns and solve potential problems, such as Simpson’s paradox^26^. Furthermore, this dataset is suitable for European or global comparisons as it comprises individual-level cases that allow for standardization.

If our dataset is supplemented with compatible information available from other sources^16,22,27^, it may prove fruitful for gearing pharmaceutical interventions (e.g., vaccination efforts and strategies). For example, a subsample of this dataset has been partly used to estimate the role of age in spreading COVID-19 across a social network in Bucharest^16,17^. The age and sex of the patients confirmed positive between August 1^st^ and October 31^st^, 2020, were input into relational hyperevent models^28,29^. Precisely, these two covariates were combined with network data (human-to-human transmission chains) to test for age and sex homophily effects^17^. Additionally, the variables embedded in our dataset may also be used, as covariates, in conjunction with network data, for estimating Exponential Random Graph Models (ERGMs)^15,30^.

Furthermore, the current dataset may prove its utility in assessing the impact of the NPIs advanced by the local authorities in Bucharest between March 7^th^ and November 11^th^, 2020. Various insights concerning the effects of the NPIs may be inferred using the sex and age covariates. For instance, the information in Table 6 implies significantly higher shares of COVID-19 cases among females when the stringency levels of the NPIs are higher. Further, the evidence exhibited in Table 6 may support the very few previous studies^31^ that claim the average age of COVID-19 patients decreases and stabilizes over time.

**Table 6:** The distribution of COVID-19 confirmed cases in Bucharest (March 7^th^ - November 11^th^, 2020), by sex and age, on weeks and phases.

Time interval	% female	average age	age std. deviation	no. of cases
Weeks 10–11	45.0	42.0	13.3	40
*Phase 1* (07.03–15.03.)	*44.7*	*40.1*	*14.4*	47
Weeks 12–13	53.6	46.3	17.5	321
Weeks 14–15	**58.4**	45.9	17.6	459
Weeks 16–17	**58.8**	47.8	16.7	403
Weeks 18–19	**57.7**	48.9	18.7	312
Weeks 20–21	**57.3**	51.0	17.7	253
*Phase 2* (16.03–14.05.)	*57.5*	*47.4*	*17.6*	*1,551*
Weeks 22–23	**61.9**	49.1	19.2	459
Weeks 24–25	**58.1**	46.0	17.7	485
*Phase 3* (15.05–16.06.)	*59.4*	*48.4*	*18.4*	932
Weeks 26–27	53.5	45.3	17.6	681
Weeks 28–29	51.4	44.1	18.2	1,059
Weeks 30–31	51.9	42.4	18.0	1,974
Weeks 32–33	51.8	43.1	18.0	2,167
Weeks 34–35	**52.6**	43.1	18.3	2,426
Weeks 36–37	51.3	43.1	17.8	2,677
Weeks 38–39	**51.8**	42.5	18.2	3,437
Weeks 40–41	**53.6**	42.7	17.8	6,307
*Phase 4* (17.06–06.10.)	*52.3*	*43.1*	*18.1*	*18,499*
Weeks 42–43	**52.9**	42.7	17.5	8,538
Weeks 44–45	**53.5**	43.6	17.3	12,045
Weeks 46–47	**53.7**	44.4	17.8	2,260
*Phase 5* (07.10–11.11.)	53.4	43.2	17.4	25,274

Calculations are made per week and phase. The number of cases in the last column does not include missing values. In the first column, statistically significant differences (p < 0.05) were marked in bold.

Our dataset may also provide a better understanding of the susceptibility to infection by biological sex. Already available scientific evidence has pointed to lower treatment efficiency^32^, greater rates of hospitalization^33^, higher probability of intensive treatment^34^, and a higher risk of death for males^35–37^. Still, the evidence is mixed when looking at confirmed cases. Earlier reports find a sex imbalance, with COVID-19 male patients having a greater risk of infection^38,39^. However, more recent studies uncover no difference between males and females regarding susceptibility to infection^40,41^.

**Fig. 4:**
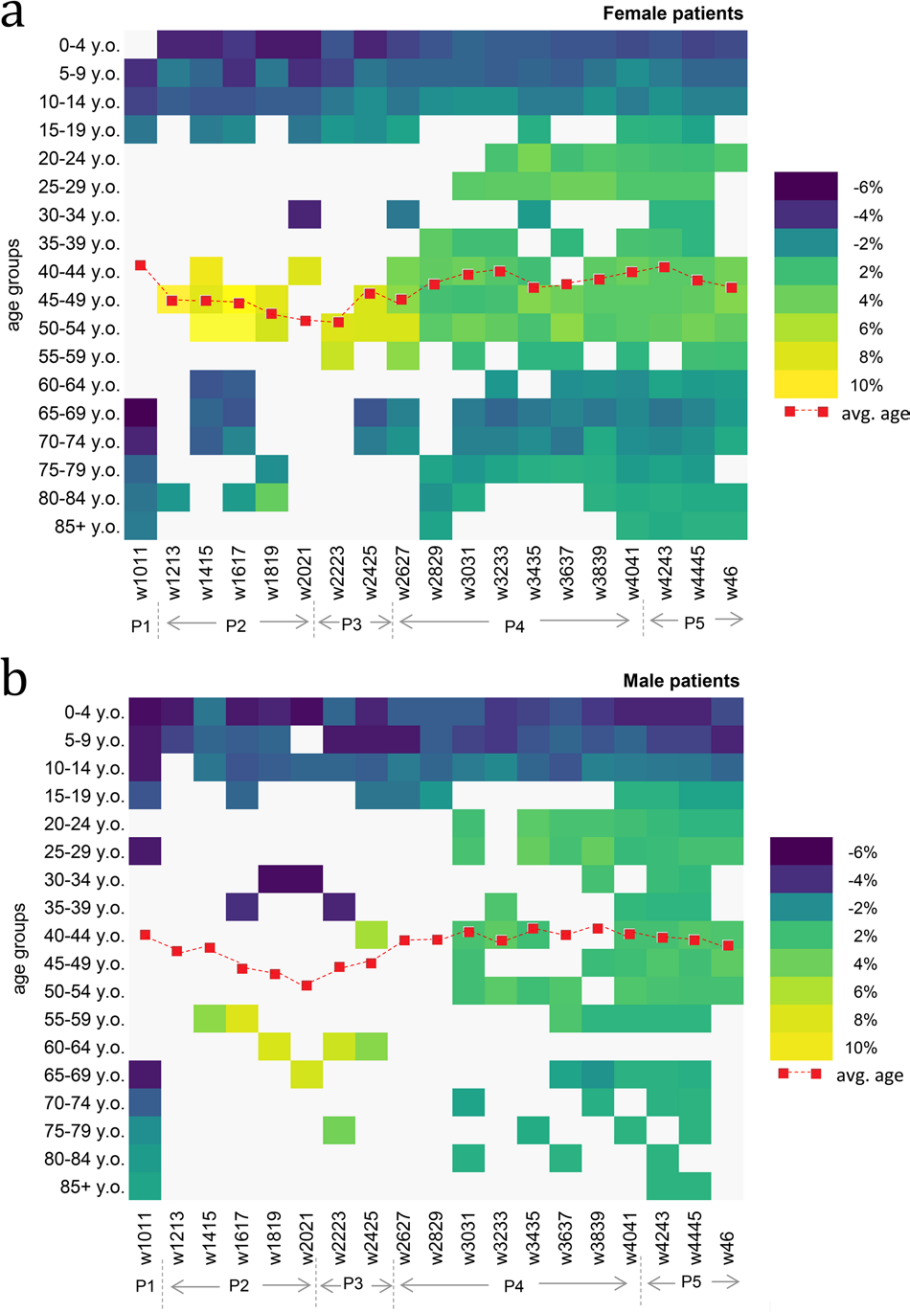
Statistically significant differences between the structure of COVID-19 confirmed cases in Bucharest and the structure of the resident population of Bucharest. We illustrate the significant differences by age groups and sex: females (**a**) and males (**b**). Brighter yellow colours designate high positive differences (more COVID-19 cases than expected when compared with the total population) and dark blue colours designate high negative differences (fewer COVID-19 cases than expected when compared with the total population).

We find in our dataset that, overall, significantly more females were confirmed with COVID-19 than males (χ^2^ = 187.64, df = 1, p = 0.000). Approximately 53.2% of all confirmed cases were females. The high level of detail in our data shows how males and females were affected during the first 250 days of the pandemic in Bucharest. For illustrative purposes, we report the differences between the Bucharest COVID-19 dataset and the resident population of Bucharest, by sex and age groups, at a 14-day time interval (Fig. 4). For females, differences range from −6.8 to +10.6, while for males, from −6.3 to +7.3. In Fig. 4, bright yellow colours designate high positive differences (more COVID-19 cases than we would expect if compared with the total population), and dark blue colours designate high negative differences (fewer COVID-19 cases than what we would expect if compared with the total population). Negative differences were found in the 0–19 age group and the 70+ age group, irrespective of the sex and confirmation date. Furthermore, the dataset provides evidence for a disproportionate impact of COVID-19 on sex, during the first few weeks of the pandemic. Throughout weeks 12 to 21, significantly high positive differences can be noticed for females aged between 40 and 54. More adult age females were tested positive during the state of emergency than we would expect compared to their share in the total resident population. The effect is not visible in the case of men. In sum, these results may reveal occupational segregation and, consequently, give support to existing reports about the unbalanced composition of the global health workforce (with females representing about 70%^42^).

Comparisons of the disaggregated COVID-19 data to population parameters are expected to have a critical role in designing health policies. The scientific community has already documented this need for demographically informed decisions, stressing the importance of interlinking the stringency and content of COVID-19 NPIs with key figures of the population^43^. For instance, school closures and curfew for individuals aged 65+ are expected to produce different outcomes depending on the structure of the population of interest. Moreover, sex and age disaggregated data are expected to guide crafting strategies for vaccination^44–46^. Last but not least, our dataset provides location details (for 83% of the cases) – see: the “district” variable, which coupled with the “confirmation date” records, could provide a spatiotemporal image for the first 250 days of the COVID-19 pandemic. In conclusion, we argue that the present disaggregated dataset can significantly improve the accuracy and effectiveness of NPIs, especially in countries with low vaccinations rates. Moreover, we deem that the qualitative scale of stringency (Fig. 1, Table 1 and the corresponding main content) is sufficiently justified and detailed that future researchers could use this, to extract, or modify it for their own purpose of exploration. Also, the modelling of COVID-19 spreading in this micro-context may be performed by corroborating our dataset with the detailed evidence available with the COVID-19 Border Accountability Project (COBAP)^47^.

## Data Availability

No codes were developed for this research.

## References

[1] Hechenbleikner EM, Samarov DV, Lin E (2020). Data explosion during COVID-19: A call for collaboration with the tech industry & data scrutiny. EClinicalMedicine.

[2] United Nations Statistics Division. SDG Indicators. *Unstats* https://unstats.un.org/sdgs/metadata/?Text=&Goal=17&Target=17.18 (2017).

[3] Global Health 50/50. Gender and sex-disaggregated data: Vital to inform an effective response to COVID-19. *Globalhealth5050* https://globalhealth5050.org/wp-content/themes/global-health/covid/media/ISSUE%20BRIEF%20-%20Sex-Disaggregated%20Data%20&%20COVID-19%20-%20Sept%202020.pdf (2020).

[4] McDougal, L. *et al*. *Strengthening Gender Measures and Data in the COVID-19 Era: An Urgent Need for Change*. (Center on Gender Equity and Health UCSD, 2021).

[5] Diaz T (2021). A call for standardised age-disaggregated health data. Lancet Healthy Longev..

[6] *European Centre for Disease Prevention and Control. COVID-19 Vaccine Tracker* https://vaccinetracker.ecdc.europa.eu/public/extensions/COVID-19/vaccine-tracker.html#uptake-tab (2022).

[7] Ritchie, H. *et al*. Coronavirus Pandemic (COVID-19). https://ourworldindata.org/covid-deaths (2022).

[8] *EURES. Labour market information. Bucharest - Romania. European Comission* https://ec.europa.eu/eures/public/living-and-working/labour-market-information/labour-market-information-romania_en (2021).

[9] *National Institute of Statistics - Romania. Usually Resident Population. Tempo Online* http://statistici.insse.ro:8077/tempo-online/#/pages/tables/insse-table (2022).

[10] Eurostat. How closely do people live together in your region? https://ec.europa.eu/eurostat/web/products-eurostat-news/-/ddn-20200430-1 (2020).

[11] Ministry of Internal Affairs. Press Release, Strategic Communication Group, March 7^th^, 2020, 5 p.m. *Ministry of Internal Affairs* https://www.mai.gov.ro/update-informare-covid-19-grupul-de-comunicare-strategica-7-martie-ora-11-00/ (2020).

[12] Hâncean M-G, Perc M, Lerner J (2020). Early spread of COVID-19 in Romania: imported cases from Italy and human-to-human transmission networks. R. Soc. Open Sci..

[13] *Code for Romania Task Force. Official Report. Stirioficiale* https://stirioficiale.ro/informatii/buletin-de-presa-21-ianuarie-2022-ora-13-00 (2022).

[14] Davies NG (2020). Age-dependent effects in the transmission and control of COVID-19 epidemics. Nat. Med..

[15] Hâncean M-G, Slavinec M, Perc M (2020). The impact of human mobility networks on the global spread of COVID-19. J. Complex Netw..

[16] Hâncean M-G (2021). Harvard Dataverse.

[17] Hâncean M-G (2021). The role of age in the spreading of COVID-19 across a social network in Bucharest. J. Complex Netw..

[18] Ministry of Internal Affairs. News. https://www.mai.gov.ro/category/comunicate-de-presa/ (2022).

[19] Ministry of Public Health. Press releases. http://www.ms.ro/comunicate (2022).

[20] *National Institute of Statistics – Romania. Tempo online* http://statistici.insse.ro:8077/tempo-online/#/pages/tables/insse-table (2022).

[21] *European Centre for Disease Prevention and Control. Daily data* https://www.ecdc.europa.eu/en/cases-2019-ncov-eueea (2022).

[22] Hâncean M-G, Perc M, Lerner J (2020). Dryad..

[23] *Permanent Electoral Authority. Vote turnout* https://prezenta.roaep.ro/locale27092020/romania-pv-final (2020).

[24] Hâncean M-G (2022). figshare.

[25] Hale T (2021). A global panel database of pandemic policies (Oxford COVID-19 Government Response Tracker). Nat. Hum. Behav..

[26] Wagner CH (1982). Simpson’s paradox in real life. Am. Stat..

[27] Hâncean M-G, Slavinec M, Perc M (2020). Zenodo.

[28] Lerner J, Lomi A (2020). Reliability of relational event model estimates under sampling: how to fit a relational event model to 360 million dyadic events. Netw. Sci..

[29] Lerner J, Lomi A, Mowbray J, Rollings N, Tranmer M (2021). Dynamic network analysis of contact diaries. Soc. Netw..

[30] Lusher, D., Koskinen, J. & Robins, G. *Exponential Random Graph Models for Social Networks: Theory, Methods, and Applications* (Cambridge University Press, 2012).

[31] Greene DN, Jackson ML, Hillyard DR, Delgado JC, Schmidt RL (2020). Decreasing median age of COVID-19 cases in the United States. Changing epidemiology or changing surveillance?. PLoS One.

[32] Mo P (2020). Clinical characteristics of refractory COVID-19 pneumonia in Wuhan, China. Clin. Infect. Dis..

[33] Klein S (2020). Biological sex impacts COVID-19 outcomes. PLoS Pathog..

[34] Iaccarino G (2020). Gender differences in predictors of intensive care units admission among COVID-19 patients: The results of the SARS-RAS study of the Italian Society of Hypertension. PLoS One.

[35] Jin JM (2020). Gender differences in patients with COVID-19: focus on severity and mortality. Front. Public Health.

[36] Vahidy FS (2021). Sex differences in susceptibility, severity, and outcomes of coronavirus disease 2019: Cross-sectional analysis from a diverse US metropolitan area. PLoS One.

[37] Huang B (2021). Sex-based clinical and immunological differences in COVID-19. BMC Infect. Dis..

[38] Guan W (2020). Clinical characteristics of coronavirus disease 2019 in China. N. Engl. J. Med..

[39] Li Q (2020). Early transmission dynamics in Wuhan, China, of novel coronavirus - infected pneumonia. N. Engl. J. Med..

[40] Peckham H (2020). Male sex identified by global COVID-19 meta-analysis as a risk factor for death and ITU admission. Nat. Commun..

[41] Gebhard C, Regitz-Zagrosek V, Neuhauser HK, Morgan R, Klein SL (2020). Impact of sex and gender on COVID-19 outcomes in Europe. Biol. Sex Diff..

[42] Boniol, M. *et al*. *Gender equity in the health workforce: Analysis of 104 countries*. WHO/HIS/HWF/Gender/WP1/2019.1 (World Health Organization, 2019)

[43] Dowd JB (2020). Demographic science aids in understanding the spread and fatality rates of COVID-19. Proc. Natl. Acad. Sci. USA.

[44] Moore S, Hill EM, Dyson L, Tildesley MJ, Keeling MJ (2021). Modelling optimal vaccination strategy for SARS-CoV-2 in the UK. PLoS Comput. Biol..

[45] Russo AG, Decarli A, Valsecchi MG (2021). Strategy to identify priority groups for COVID-19 vaccination: A population based cohort study. Vaccine.

[46] Hsieh YH (2010). Age groups and spread of influenza: implications for vaccination strategy. BMC Infect. Dis..

[47] Shiraef MA (2021). COVID Border accountability project, a hand-coded global database of border closures introduced during 2020. Sci. Data.

